# Impact of activation energy and variable properties on peristaltic flow through porous wall channel

**DOI:** 10.1038/s41598-023-30334-3

**Published:** 2023-02-24

**Authors:** Maimona Rafiq, Asma Shaheen, Youssef Trabelsi, Sayed M. Eldin, M. Ijaz Khan, Dhia Kadhm Suker

**Affiliations:** 1 Department of Mathematics, COMSATS University Islamabad, Attock, 43600 Pakistan; 2 Department of Physics, College of Science and Arts at Muhayel, King Khalid University, Abha, Saudi Arabia; 3 Center of Research, Faculty of Engineering, Future University in Egypt New Cairo, New Cairo, 11835 Egypt; 4 Department of Mechanical Engineering, Lebanese American University, Beirut, Lebanon; 5 Department of Mathematics and Statistics, Riphah International University, I-14, Islamabad, 44000 Pakistan; 6 Mechanical Engineering Department, College of Engineering and Islamic Architecture, Umm Al-Qura University, P. O. Box 5555, Mecca, 21955 Saudi Arabia

**Keywords:** Biophysics, Physiology, Mathematics and computing

## Abstract

The current study discusses the peristaltic flow of Jeffrey fluid through a porous wall channel. Magnetohydrodynamic (MHD) effects are also considered while formulating the problem. Heat and mass transfers are discussed in the presence of activation energy and constant heat source/sink effects. A chemical reaction is also part of the analysis. The Lubrication approach is adopted for the simplification of resulting non-linear equations. MATHEMATICA command, NDSolve, is used to discuss the results graphically for various flow parameters like Hartman number $(M)$, porosity parameter $(k)$, slip parameters ($\gamma ,{\gamma }_{1},{\gamma }_{2}$), Schmidt $(Sc)$, Soret $(Sr)$ and Prandtl $(Pr)$ numbers, and many others. Parabolic behavior for velocity and sinusoidal nature for heat transfer and pressure gradient is noticed. Results indicate that the velocity is greatly affected by varying values of slip parameters (*γ*′*s*) and Hartman number $(H)$. Enhancing the viscoelastic nature of fluid causes an increase in velocity. Similar behavior is noticed for velocity and temperature profiles. The decreasing trend is shown by concentration when the value of the chemical reaction and temperature ratio parameters is enhanced. Thus, the study presented in the current analysis can be used to study many human physiological systems especially, the blood flow. Since Jeffrey's fluid exhibits the same characteristics as observed for blood.

## Introduction

Mathematical modeling is employed in biomechanics to investigate the physiological systems. Bio-fluid mechanics is a section of biomechanics which reveals the kinematics and dynamics of body fluids in living beings. Advancement in bio-fluid mechanics enable scientists to study the blood vessels liquid stream, the respiratory tract, the lymphatic system, the gastrointestinal tract, the urinary tract and various other. Recent investigations disclose the clinical applications like artificial organs, vascular vessel advancement, designing medical instruments, creation of material membranes for orthopedics, and many more. Analogous processes of bio-liquids flow can be seen in variety of situations within human body among which the prominent is peristalsis and can be seen as basis for the current study. The main purpose of peristalsis is to move the fluids through the tubular structure without requiring an overall pressure difference. The term peristalsis originates from the Greek word peristaltilkos, meaning “compressing and clasping”. According to Merriam-Webster’s^1^ peristalsis is consecutive waves of involuntary contraction passing along the walls of a hollow muscular structure and pushing the contents forward. The human body's peristalsis mechanism started working after food was chewed, swallowed as a bolus, and passed through the oesophagus. To prevent the bolus from voyaging back toward the mouth, smooth muscles behind it constrict. It was first described as a kind of motility where there is contraction above and relaxation below a transported by Bayliss and Starling^2^. Industrial application of peristaltic pumping is exploited in different applications which include sterile fluids exchange, blood pump in heart lung machines, transportation of internecine and dangerous liquids to prevent their involvement in surrounding environment etc. A noteworthy modern use of peristaltic pumping can be seen in designing the roller pumps which are used to avoid the contact of fluid with the pumping equipment. Peristaltic transport on viscous liquids was first introduced by Latham^3^, in 1966. This study was further extended by Shapiro et al.^4^.

In fact, not every fluid possesses the characteristics of a Newtonian fluid. Therefore, we include non-Newtonian fluids in our discussion. However, realistically speaking, complicated fluids like food boluses travelling through the oesophagus, urine passing through the ureter, or chyme traversing the gastrointestinal track would not adhere to Newton's principles of viscosity. As a result, no single constitutive relation can predict the characteristics of all fluids. In response to this issue, a number of constitutive models have been proposed to identify the properties of non-Newtonian fluids. The equations for the flow of pigment oil suspensions used in printing ink-type fluid were expanded by Casson^5^. The theory of the micro-polar fluid was covered by Eringen^6^, who also thoroughly investigated features such couple stresses, body couples, micro-rotation, and micro inertial effects. The theory of micro-fluids, of which micro-polar is a specific instance, was first presented by Eringen^6^. Among all of these models, the Jeffrey fluid, which possesses both relaxing and retarding qualities, is comparatively one of the simplest types of viscoelastic fluid. Because Jeffrey fluid can forecast relaxation/retardation time effects, which are crucial for the analysis of viscoelastic properties in the polymer industries and human physiological systems. Ramanamurthy et al.^7^ investigated the peristaltic flow of viscous fluid through two-dimensional curved channel. Their main aim is to analyze unsteady nature of the flow. Nadeem et al.^8^ performed the endoscope analysis of Prandtl liquid in both fixed and wave frame of references. They have addressed the effect of various wave shapes on endoscope. Power-law fluid flow through cylindrical tube is examined by Sadeghi and Talab^9^. The results indicate enhancement in the fluid flow due to high values of power-law index. Tripathi et al.^10^ developed mathematical model to discuss intestinal flow by taking bi-viscosity non-Newtonian fluid. Their study helps in better understanding of gastric-hydrodynamics. Axisymmetric flow of Bingham fluid through cylindrical geometry is investigated by Fusi and Farina^11^. Ramesh and Devaker^12^ modeled endoscope problem to discuss its application to biomedicine. They have taken couple stress fluid to model physiological fluid. Application of peristalsis to chyme movement in gastrointestinal tract can be seen through Vaidya et al.^13^. Their study discloses the increasing impact of variable viscosity on bolus size. In the culmination of above mentioned literature, we can observe the real life applications of non-Newtonian fluid flow in medical and industry.

The study of magneto-hydro-dynamics is concerned with the motion of highly conducting fluids in the presence of a magnetic field. The velocity of the conducting fluid through magnetic field generates electric current that change the magnetic field and generates mechanical forces that change the fluid flow^14^. Due to its substantial applications, such as in materials processing, magneto-hydro-dynamic (MHD) energy generators^15^, cancer therapy^16^, and biomedical flow control and separation devices^17^, the impact of bio-magnetic fluid dynamics has attained a prominent position. Its use in biomedical engineering includes magneto-fluid rotary blood pumps, MHD medication targeting, and controlling hyperthermia in the cardiovascular system^18,19^. A device that uses a magnetic field and a highly sensitive sensor to detect minute movements of an object within the magnetic field is known as a giant magneto resistive (GMR) device. Research into peristaltic activity inside tubular organs like the colon, fallopian tubes, and even the vas deferens has improved because to this technique. Satyanarayana et al.^20^ explores the impact of magnetic field on peristaltic flow of micropolar fluid through asymmetric channel. Their study reveals that velocity increases with the enhancement in micro-rotaion parameter. Peristaltic flow with variable thermal conductivity and viscosity is addressed by Latif et al.^21^. Their study concluded that Newtonian fluid has low heat transfer rate as compared to third order fluid. Prakash et al.^22^ considered Williamson fluid to model blood flow in the presence of magnetic field. Selvi and Sirinivas^23^ addresses the peristaltic transport of Herschel-Bulkley fluid through non-uniform tube. They verify their results by comparing them to Vajravelu et al. Shera et al.^24^ worked on mathematical analysis of thermal therapy used for the treatment of cancer.

Heat transfer plays vital role in industrial and medical applications. Especially, within human body heat transfer is an essential area of research. Bio-heat transfer in tissues has attracted the attention of biomedical engineers for thermotherapy^25^ and the human thermoregulation system^26^. The heat transfer inside human body occurs as conduction in tissues, perfusion of the arterial-venous blood through the pores of the tissue, metabolic heat generation whereas the annihilation of cancerous cells, dilution technique of blood flow and vasodilation. In connection with peristalsis heat transfer become significant in oxygenation and hemodialysis. Several researchers investigated about heat transfer in peristaltically induced flows. In general, viscous dissipation effect is ignored while performing theoretical analysis of fluid flow problems. But under certain situations this supposition may lead to doubtful results. The need of considering viscous dissipation effects is felt while dealing with strong temperature-dependent viscosity, high viscosity fluids and high-speed gas dynamics. Heat produced due to viscous dissipation may increase the temperature of tube wall consequently decreasing viscosity which results in increased velocity and temperature. Thus, instead of viscous dissipation effects variable thermal conductivity is considered to make analysis more realistic. While discussing heat transfer, we cannot ignore mass transfer phenomenon as simultaneous occurrence of both can be seen through many applications such as drying, energy transfer in wet cooling tower, evaporation at the surface of a water body and the ow in dessert cooler. Additionally, mass transfer manifests as a result of the different species' concentrations in a combination liquid. Such characteristics change as a mixture is transported from areas of higher to lower concentration. Additionally, activation energy, which is referred to as the least obligatory energy that chemical reactants must gain before the chemical reaction takes place, is one of the most crucial characteristics for chemical reactants. Chemical engineering, geothermal reservoirs, oil emulsions, and water mechanics, among other fields, all heavily depend on the consideration of mass transfer with both chemical reaction and activation energy. Tanveer et al. presented the analysis for electroosmotic peristaltic flow of nanofluid with non-Newtonian base fluid (see refs.^27,28^). The study focuses the attention on thermal aspects of the flow. Thus, finding promising applications in micro-fabrication and chemical industry. Maryam et al.^29^ presented the meta-analysis of homogeneous–heterogeneous chemical reaction on peristaltic flow through wavy curved geometry. Ahmed et al.^30^ studies thermal radiation effects on peristaltic flow of nanofluid with mixed convection. The study unveils that the magnetic field tends to increase the thermal energy of the flow. Microfluidic peristaltic flow is examined by Noreen et al.^31^ in view of heat transfer and electroosmotic effects. Convective heat and mass transfer for Prandtl nanofluid through non-uniform channel is analyzed by Akram et al.^32^. Khazayinejad et al.^33^ presented mathematical model for the treatment of cancer by injecting graphene particles in to blood stream and then, applying external magnetic field. Nanoparticle volume fraction enhancement results in greater heat transfer. Hence, shows great impact on the destruction of cancerous cells. Imran et al.^34–36^ discusses effect of various types of chemical reactions on peristaltic flow through various geometries. Analysis of physiological liquid flow between absorbent barriers, such as blood, became crucial, particularly in the lungs. According to Fung and Tang^37^ and Gopalan^38^, the lung can be seen as a duct encircled by two thin, porous media layers. Therefore, other researchers like Naveed et al.^39,40^ presented mathematical formulation of different non-Newtonian fluids through porous medium as real life application of peristalsis. Some other important and meaningful research on miscellaneous disciplines i.e., nanoribbons, non-uniform wall thickness, granular thermodynamics framework^41–43^, material analysis^44–46^, fluid flow and mass transport in multi-scale heterogeneous media and porosity effects^47–50^. Saima et al.^51^ focus their attention on the heat and mass transfer analysis of micro-polar nanofluid through a lid driven cavity. They adopted finite element method to solve the obtained nonlinear system of equations. The results indicate that great mass diffusion occurs inside the cavity for small Schmidt number. Rasool et al.^52^ presented the study for thermal analysis of Maxwell nanofluid over isothermally heated surface. Their study is based on the comparison on convective and non-convective boundary conditions. In addition, entropy generation analysis of multi-walled carbon nanoparticles (MWCN) inside vertical Cleaveland Z-staggered cavity is also performed by Rassol et al.^53^. The outcome of the study shows that for higher Reynolds number Bejan number decreases creating great impact on entropy generation. Investigation of elrctro-magneto-hydrodynamic nanofluid flow over riga plate is investigated in^54^. The study unveils that the heat transfer across the wall can be controlled by adjusting convective conditions.

The flow field can be dramatically altered by the suction or injection of fluid through the bounding surfaces, as in mass transfer cooling, which then affects the rate of heat transfer from the bounding surfaces. In general, injection functions in the opposite ways of suction, which tends to enhance skin-friction and heat transfer coefficients. Fluid injection or withdrawal via porous heated or cooled surfaces is generally of importance in real-world issues like blood flow through arteries, fluid flow through urinary tract etc. This may result in the system being heated (or cooled) more effectively. Moreover, viscoelastic nature of blood can be exactly exhibited by the considered Jeffrey fluid model. Also, different types of chemical reactions within human physiological systems, particularly during blood flow, greatly affect the fluid flows. Therefore, giving consideration to such effects provide us with more realistic mathematical model of various physiological systems. Therefore, goal of this study is to develop a mathematical model for Jeffrey fluid that incorporates activation energy and peristaltic flow via a porous walled channel. In order to adjust the MHD flow brought on by peristalsis, the presence of porous walled channels and the effects of activation energy on non-Newtonian fluid will be crucial. The construction of nonlinear coupled equations via mathematical modelling will be discussed, and the lubrication approach will then be used to simplify them. The plotting of graphs will be used to explain the effects of applicable factors and parameters on the flow via built-in NDSolve command of MATHEMATICA.

## Mathematical model

**Figure 1:**
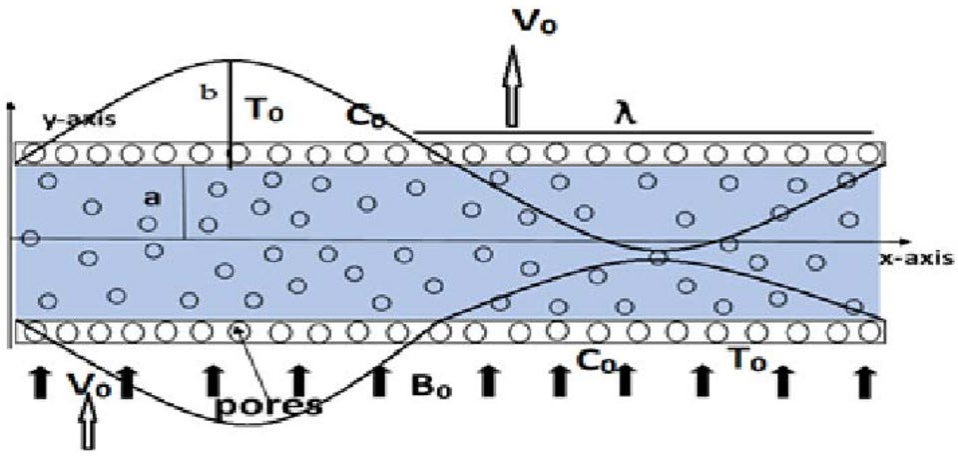
Geometry of the problem.

Through a flexible walled conduit, we examine the incompressible 2D peristaltic flow of Jeffrey fluid. The walls of the channels are permeable. A magnetic field is taken that is parallel to the flow. Sinusoidal waves with a wavelength of λ are moving at a speed of $c$ along the walls. Let $u$ and $v$ represent the axial and transverse velocity components, respectively. The Cartesian coordinate system is used to discuss the flow (see Fig. 1).

## Mathematical formulation

Wall surface geometry mathematical expression is expressed as under^3,4^:

1 $$\overline{{\varvec{H}} }\left(\overline{{\varvec{X}} },\overline{{\varvec{t}} }\right)={\varvec{a}}+{\varvec{b}}{\varvec{S}}{\varvec{i}}{\varvec{n}}\left[\frac{2{\varvec{\pi}}}{{\varvec{\lambda}}}\left(\overline{{\varvec{X}} }-{\varvec{c}}\overline{{\varvec{t}} }\right)\right],$$

where $\overline{t }$ denotes time, $\lambda$ wavelength, $b$ wave amplitude, $a$ width of channel, $c$ is the wave speed and $\overline{X }$ is the horizontal direction. The governing equations for the flow are^55,57–59^:

2 $$\frac{\partial \overline{{\varvec{U}}} }{\partial \overline{{\varvec{X}}} }+\frac{\partial \overline{{\varvec{V}}} }{\partial \overline{{\varvec{Y}}} }=0 ,$$

3 $${\varvec{\rho}}\left[\frac{\partial \overline{{\varvec{U}}} }{\partial \overline{{\varvec{t}}} }+\overline{{\varvec{U}} }\frac{\partial \overline{{\varvec{U}}} }{\partial \overline{{\varvec{X}}} }+\overline{{{\varvec{V}} }_{0}}\frac{\partial \overline{{\varvec{V}}} }{\partial \overline{{\varvec{Y}}} }\right]=-\frac{\partial \overline{{\varvec{P}}} }{\partial \overline{{\varvec{X}}} }+\frac{\partial }{\partial \overline{{\varvec{X}}} }\left({{\varvec{\tau}}}_{\overline{{\varvec{X}} }\overline{{\varvec{X}}} }\right)+\frac{\partial }{\partial \overline{{\varvec{Y}}} }\left({{\varvec{\tau}}}_{\overline{{\varvec{X}} }\overline{{\varvec{Y}}} }\right)-{\varvec{\sigma}}{{\varvec{B}}}_{0}^{2}\overline{{\varvec{U}}},$$

4 $${\varvec{\rho}}\left[\frac{\partial \overline{{\varvec{V}}} }{\partial \overline{{\varvec{t}}} }+\overline{{\varvec{U}} }\frac{\partial \overline{{\varvec{V}}} }{\partial \overline{{\varvec{X}}} }+\overline{{{\varvec{V}} }_{0}}\frac{\partial \overline{{\varvec{V}}} }{\partial \overline{{\varvec{Y}}} }\right]=-\frac{\partial \overline{{\varvec{P}}} }{\partial \overline{{\varvec{Y}}} }+\frac{\partial }{\partial \overline{{\varvec{X}}} }\left({{\varvec{\tau}}}_{\overline{{\varvec{Y}} }\overline{{\varvec{X}}} }\right)+\frac{\partial }{\partial \overline{{\varvec{Y}}} }\left({{\varvec{\tau}}}_{\overline{{\varvec{Y}} }\overline{{\varvec{Y}}} }\right) ,$$

5 $$\rho {C}_{p}\left[\frac{\partial \overline{T} }{\partial \overline{t} }+\overline{U }\frac{\partial \overline{T} }{\partial \overline{X} }+\overline{{V }_{0}}\frac{\partial \overline{T} }{\partial \overline{Y} }\right]=\overline{K }\left(\overline{T }\right)\left[\frac{{\partial }^{2}\overline{T} }{\partial \overline{{X }^{2}}}+\frac{{\partial }^{2}\overline{T} }{\partial \overline{{Y }^{2}}}\right]+{Q}_{0} ,$$

6 $$\left[\frac{\partial \overline{C} }{\partial \overline{t} }+\overline{U }\frac{\partial \overline{C} }{\partial \overline{X} }+\overline{{V }_{0}}\frac{\partial \overline{C} }{\partial \overline{Y} }\right]=D\left[\frac{{\partial }^{2}\overline{C} }{\partial \overline{{X }^{2}}}+\frac{{\partial }^{2}C}{\partial \overline{{Y }^{2}}}\right]-{K}_{1}\left(C-{C}_{0}\right)+{K}_{2}^{2}{\left(\frac{\overline{T} }{\overline{{T }_{1}}-{\overline{T} }_{0}}\right)}^{n}{e}^{\frac{{E}_{a}}{{K}^{*}\overline{T}} }\left(C-{C}_{0}\right) ,$$

where shear stress components are given as^60^:

7 $${{\varvec{\tau}}}_{\overline{{\varvec{X}} }\overline{{\varvec{X}}} }=\frac{2{\varvec{\mu}}}{(1+{{\varvec{\uplambda}}}_{1})}\left(1+\overline{{{\varvec{\lambda}} }_{2}}\left(\overline{{\varvec{U}}}\frac{\partial }{\partial \overline{{\varvec{X}}} }+\overline{{\varvec{V}}}\frac{\partial }{\partial \overline{{\varvec{Y}}} }\right)\right)\frac{\partial \overline{{\varvec{U}}} }{\partial \overline{{\varvec{X}}}},$$

8 $${{\varvec{\tau}}}_{\overline{{\varvec{X}} }\overline{{\varvec{Y}}} }=\frac{{\varvec{\mu}}}{(1+{{\varvec{\uplambda}}}_{1})}\left(1+\overline{{{\varvec{\lambda}} }_{2}}\left(\overline{{\varvec{U}}}\frac{\partial }{\partial \overline{{\varvec{X}}} }+\overline{{\varvec{V}}}\frac{\partial }{\partial \overline{{\varvec{Y}}} }\right)\right)\left(\frac{\partial \overline{{\varvec{U}}} }{\partial \overline{{\varvec{Y}}} }+\frac{\partial \overline{{\varvec{V}}} }{\partial \overline{{\varvec{X}}} }\right),$$

9 $${{\varvec{\tau}}}_{\overline{{\varvec{Y}} }\overline{{\varvec{Y}}} }=\frac{2{\varvec{\mu}}}{(1+{{\varvec{\uplambda}}}_{1})}\left(1+\overline{{{\varvec{\lambda}} }_{2}}\left(\overline{{\varvec{U}}}\frac{\partial }{\partial \overline{{\varvec{X}}} }+\overline{{\varvec{V}}}\frac{\partial }{\partial \overline{{\varvec{Y}}} }\right)\right)\frac{\partial \overline{{\varvec{V}}} }{\partial \overline{{\varvec{Y}}}},$$

where variable thermal conductivity is taken as^56^:

10 $$\overline{{\varvec{K}} }\left(\overline{{\varvec{T}} }\right)={{\varvec{K}}}_{{\varvec{m}}}\left(1+\boldsymbol{\alpha }\left(\frac{\overline{{\varvec{T}} }-{{\varvec{T}}}_{0}}{{{\varvec{T}}}_{1}-{{\varvec{T}}}_{0}}\right)\right).$$

The boundary conditions for the analysis are considered as^26,27^:

11 $$\frac{\partial \overline{{\varvec{U}}} }{\partial \overline{{\varvec{Y}}} }=0\;{\varvec{at}}\; \overline{{\varvec{Y}} }=0,$$

12 $$\overline{{\varvec{U}} }+\overline{{\varvec{\gamma}} }\frac{\partial \overline{{\varvec{U}}} }{\partial \overline{{\varvec{Y}}} }=0\; {\varvec{at}}\; \overline{{\varvec{Y}} }=\overline{{\varvec{H}} },$$

13 $$\frac{\partial \overline{{\varvec{T}}} }{\partial \overline{{\varvec{Y}}} }=0\; {\varvec{at}}\; \overline{{\varvec{Y}} }=0,$$

14 $$\overline{{\varvec{T}} }+\overline{{{\varvec{\gamma}} }_{1}}\frac{\partial \overline{{\varvec{T}}} }{\partial \overline{{\varvec{Y}}} }={{\varvec{T}}}_{0}\; {\varvec{at}}\; \overline{{\varvec{Y}} }=\overline{{\varvec{H}} },$$

15 $$\frac{\partial \overline{{\varvec{C}}} }{\partial \overline{{\varvec{Y}}} }=0\; {\varvec{at}}\; \overline{{\varvec{Y}} }=0,$$

16 $$\overline{{\varvec{C}} }+\overline{{{\varvec{\gamma}} }_{2}}\frac{\partial \overline{{\varvec{C}}} }{\partial \overline{{\varvec{Y}}} }={{\varvec{C}}}_{0}\; {\varvec{at}}\; \overline{{\varvec{Y}} }=\overline{{\varvec{H}} }.$$

In above equations, $\overline{U }$ and $\overline{V }$ stands for velocity components in $\overline{X }$ and $\overline{Y }$ directions respectively whereas ρ denotes density. $\overline{{V }_{0}}$ is the velocity through porous walls, $\overline{P }$ is the pressure and shows electrical conductivity. $\overline{{B }_{0}}$ is magnetic field strength and specific heat capacity is shown by ${C}_{p}$. $D$ is mass diffusion coefficient and ${Q}_{0}$ is heat source/sink parameter. ${K}_{1}$ is chemical reaction parameter and ${K}_{2}$ is the reaction rate. ${E}_{a}$ stands for activation energy, $n$ is the fitted rate constant and ${K}^{*}$ is Boltzmann constant, ${K}_{m}$ be the thermal conductivity at constant temperature and α be the constant. $\overline{\gamma }$, $\overline{{\gamma }_{1}}$ and $\overline{{\gamma }_{2}}$ are velocity, temperature and concentration slip parameters respectively.

The transformation between steady and unsteady reference frames is

17 $$\overline{y} = \overline{Y},\;\overline{x} = - c\overline{t} + \overline{X} ,\;\overline{u} = - c + \overline{U},\;\overline{p}\left( {\overline{x},\overline{y}} \right) = \overline{P}\left( {\overline{X},\overline{Y}} \right),\;\overline{{v_{0} }} = \overline{{V_{0} }} ,$$

where $\overline{{V_{0} }}$. is the suction/injection parameter at the walls. The dimensionless constants/variables are defined as:

18 $$\begin{aligned} {\varvec{x}} & = \frac{{\overline{\user2{x}}}}{{\varvec{\lambda}}},\user2{ y} = \frac{{\overline{\user2{y}}}}{{\varvec{a}}},\user2{ p} = \frac{{{\varvec{a}}^{2} \overline{\user2{p}}}}{{\user2{c\mu \lambda }}},\user2{ t} = \frac{{\user2{c\overline{t}}}}{{\varvec{\lambda}}},{\varvec{u}} = \frac{{\overline{\user2{u}}}}{{\varvec{c}}},\user2{ v}_{0} = \frac{{\overline{{{\varvec{v}}_{0} }} }}{{\user2{c\delta }}},\user2{ \tau }_{{{\varvec{ij}}}} = \frac{{{\varvec{a}}\overline{{{\varvec{\tau}}_{{{\varvec{ij}}}} }} }}{{\user2{\mu c}}},\user2{ h} = \frac{{\overline{\user2{H}}}}{{\varvec{a}}}\user2{ },\user2{ K}_{{\varvec{c}}} = \frac{{{\varvec{K}}_{1} {\varvec{a}}^{2} }}{{\varvec{\nu}}} \\ {\varvec{\delta}} & = \frac{{\varvec{a}}}{{\varvec{\lambda}}}\user2{ },\user2{ \lambda }_{2} = \frac{{\overline{{{\varvec{\lambda}}_{2} }} {\varvec{c}}}}{{\varvec{a}}}\user2{ },\user2{ Pr} = \frac{{\user2{\mu C}_{{\varvec{p}}} }}{{{\varvec{K}}_{{\varvec{m}}} }}\user2{ },\user2{ R}_{{\varvec{e}}} = \frac{{{\varvec{ca}}}}{{\varvec{\nu}}}\user2{ },\user2{ Sc} = \frac{{\varvec{\nu}}}{{\varvec{D}}}\user2{ },\user2{ H} = \sqrt {\frac{{\varvec{\sigma}}}{{\varvec{\mu}}}} {\varvec{B}}_{0} \user2{a },\user2{ B} = \frac{{{\varvec{Q}}_{0} {\varvec{a}}^{2} }}{{{\varvec{\nu}}\left( {{\varvec{T}}_{1} - {\varvec{T}}_{0} } \right)}}\user2{ }, \\ {\varvec{\gamma}}_{{\varvec{i}}} & = \frac{{\overline{{{\varvec{\gamma}}_{{\varvec{i}}} }} }}{{\varvec{a}}}\user2{ }\left( {{\varvec{i}} = 0,1,2} \right),\user2{ \theta } = \frac{{\overline{\user2{T}} - {\varvec{T}}_{0} }}{{{\varvec{T}}_{1} - {\varvec{T}}_{0} }},\user2{ }\phi = \frac{{\overline{\user2{C}} - {\varvec{C}}_{0} }}{{{\varvec{C}}_{1} - {\varvec{C}}_{0} }},\user2{ E} = \frac{{{\varvec{E}}_{{\varvec{a}}} }}{{{\varvec{K}}^{\user2{*}} {\varvec{T}}_{0} }}\user2{ },{\varvec{\xi}} = \frac{{{\varvec{K}}_{1} {\varvec{d}}^{2} }}{{\varvec{\nu}}}\user2{ },\user2{ \Omega } = \frac{{{\varvec{T}}_{1} - {\varvec{T}}_{0} }}{{{\varvec{T}}_{0} }}\user2{ }. \\ \end{aligned}$$

After using the above transformation, dimensional equations take the form

19 $${\varvec{\delta}}\frac{\partial {\varvec{u}}}{\partial {\varvec{x}}}+\frac{\partial {\varvec{v}}}{\partial {\varvec{y}}}=0,$$

20 $${\varvec{R}}{\varvec{e}}\left[({\varvec{u}}+1){\varvec{\delta}}\frac{\partial {\varvec{u}}}{\partial {\varvec{x}}}+{{\varvec{v}}}_{0}\frac{\partial {\varvec{u}}}{\partial {\varvec{y}}}\right]=-\frac{\partial {\varvec{p}}}{\partial {\varvec{x}}}+{\varvec{\delta}}\frac{\partial }{\partial {\varvec{x}}}\left({{\varvec{\tau}}}_{{\varvec{x}}{\varvec{x}}}\right)+\frac{\partial }{\partial {\varvec{y}}}\left({{\varvec{\tau}}}_{{\varvec{x}}{\varvec{y}}}\right)-{{\varvec{H}}}^{2}({\varvec{u}}+1),$$

21 $${\varvec{R}}{\varvec{e}}{\varvec{\delta}}\left[({\varvec{u}}+1){\varvec{\delta}}\frac{\partial {\varvec{v}}}{\partial {\varvec{x}}}+{{\varvec{v}}}_{0}\frac{\partial {\varvec{v}}}{\partial {\varvec{y}}}\right]=-\frac{\partial {\varvec{p}}}{\partial {\varvec{y}}}+{{\varvec{\delta}}}^{2}\frac{\partial }{\partial {\varvec{x}}}\left({{\varvec{\tau}}}_{{\varvec{y}}{\varvec{x}}}\right)+\frac{\partial }{\partial {\varvec{y}}}\left({{\varvec{\tau}}}_{{\varvec{y}}{\varvec{y}}}\right),$$

22 $${\varvec{R}}{\varvec{e}}{{\varvec{v}}}_{0}\left[({\varvec{u}}+1){\varvec{\delta}}\frac{\partial{\varvec{\theta}}}{\partial {\varvec{x}}}+{{\varvec{v}}}_{0}\frac{\partial{\varvec{\theta}}}{\partial {\varvec{y}}}\right]=\frac{{\varvec{K}}({\varvec{\theta}})}{{\varvec{P}}{\varvec{r}}}\left[{{\varvec{\delta}}}^{2}\frac{{\partial }^{2}{\varvec{\theta}}}{\partial {{\varvec{x}}}^{2}}+\frac{{\partial }^{2}{\varvec{\theta}}}{\partial {{\varvec{y}}}^{2}}\right]+{\varvec{B}},$$

23 $${\varvec{R}}{\varvec{e}}{{\varvec{v}}}_{0}{\varvec{S}}{\varvec{c}}\frac{\partial{\varvec{\phi}}}{\partial {\varvec{y}}}+{\varvec{S}}{\varvec{c}}{\varvec{\delta}}\left({\varvec{u}}+1\right)\frac{\partial{\varvec{\phi}}}{\partial {\varvec{x}}}=\left[{{\varvec{\delta}}}^{2}\frac{{\partial }^{2}{\varvec{\phi}}}{\partial {{\varvec{x}}}^{2}}+\frac{{\partial }^{2}{\varvec{\phi}}}{\partial {{\varvec{y}}}^{2}}\right]-{\varvec{S}}{\varvec{c}}{{\varvec{K}}}_{{\varvec{c}}}{\varvec{\phi}}+{\varvec{\xi}}{\varvec{S}}{\varvec{c}}{\varvec{\phi}}\left(\boldsymbol{\Omega }{\varvec{\theta}}+1\right){{\varvec{e}}}^{{\varvec{E}}{\left(\boldsymbol{\Omega }{\varvec{\theta}}+1\right)}^{-1}},$$

and shear stress components are given as:

24 $${\varvec{\tau}}_{{{\varvec{xx}}}} = \user2{ }\frac{{2{\varvec{\delta}}}}{{\left( {1 + {{\varvec{\uplambda}}}_{1} } \right)}}\left( {\frac{{\partial {\varvec{u}}}}{{\partial {\varvec{x}}}} + {{\varvec{\uplambda}}}_{2} \left( {{\varvec{\delta}}\left( {{\varvec{u}} + 1} \right)\frac{\partial }{{\partial {\varvec{x}}}} + {\varvec{v}}_{0} \frac{\partial }{{\partial {\varvec{y}}}}} \right)\frac{{\partial {\varvec{u}}}}{{\partial {\varvec{x}}}}} \right)\user2{ },$$

25 $${\varvec{\tau}}_{{{\varvec{xy}}}} = \user2{ }\frac{2}{{\left( {1 + {{\varvec{\uplambda}}}_{1} } \right)}}\left( {1 + {{\varvec{\uplambda}}}_{2} \left( {{\varvec{\delta}}\left( {{\varvec{u}} + 1} \right)\frac{\partial }{{\partial {\varvec{x}}}} + {\varvec{v}}_{0} \frac{\partial }{{\partial {\varvec{y}}}}} \right)\frac{{\partial {\varvec{u}}}}{{\partial {\varvec{x}}}}} \right)\left( {\frac{{\partial {\varvec{u}}}}{{\partial {\varvec{y}}}} + {\varvec{\delta}}\frac{{\partial {\varvec{v}}_{0} }}{{\partial {\varvec{y}}}}} \right)\user2{ },$$

26 $${\varvec{\tau}}_{{{\varvec{yy}}}} = \user2{ }\frac{2}{{\left( {1 + {{\varvec{\uplambda}}}_{1} } \right)}}\left( {\frac{{\partial {\varvec{v}}_{0} }}{{\partial {\varvec{y}}}} + {{\varvec{\uplambda}}}_{2} \left( {{\varvec{\delta}}\left( {{\varvec{u}} + 1} \right)\frac{\partial }{{\partial {\varvec{x}}}} + {\varvec{v}}_{0} \frac{\partial }{{\partial {\varvec{y}}}}} \right)\frac{{\partial {\varvec{v}}_{0} }}{{\partial {\varvec{y}}}}} \right)\user2{ },$$

where $\delta$ is the wave number, $Re$ the Reynolds number, $H$ stands for Hartman number, $Pr$ is the Prandtl number whereas $B$ shows constant heat source/sink parameter. $Sc$ exhibits Schmidt number, ${K}_{c}$ is chemical reaction parameter, $\Omega$ is temperature ratio parameter, $E$ the activation energy parameter whereas $n$ is the fitted rate constant. Employing long wavelength approximation to above equations, we get:

27 $$\frac{\partial {\varvec{v}}}{\partial {\varvec{y}}}=0,$$

28 $$\frac{1}{(1+{{\varvec{\uplambda}}}_{1})}\frac{{\partial }^{2}{\varvec{u}}}{\partial {{\varvec{y}}}^{2}}-{\varvec{k}}\frac{\partial {\varvec{u}}}{\partial {\varvec{y}}}-{\varvec{P}}-{{\varvec{H}}}^{2}\left({\varvec{u}}+1\right)=0 ,$$

29 $$-\frac{\partial {\varvec{p}}}{\partial {\varvec{y}}}=0,$$

30 $$\frac{\partial }{\partial {\varvec{y}}}\left(1+\boldsymbol{\alpha }{\varvec{\theta}}\right)\frac{{\partial }^{2}{\varvec{\theta}}}{\partial {{\varvec{y}}}^{2}}-{\varvec{P}}{\varvec{r}}{\varvec{k}}\frac{\partial{\varvec{\theta}}}{\partial {\varvec{y}}}+{\varvec{P}}{\varvec{r}}{\varvec{B}}=0,$$

31 $$\frac{{\partial }^{2}\phi }{\partial {y}^{2}}-kSc\frac{\partial \phi }{\partial y}-Sc{K}_{c}\phi +\xi Sc\phi \left(\Omega \theta +1\right){e}^{E{\left(\Omega \theta +1\right)}^{-1}}=0 ,$$

and the shear stress components become:

32 $${{\varvec{\tau}}}_{{\varvec{x}}{\varvec{x}}}=0,$$

33 $${{\varvec{\tau}}}_{{\varvec{x}}{\varvec{y}}}=\frac{2}{(1+{{\varvec{\uplambda}}}_{1})}\left(1+{{\varvec{\uplambda}}}_{2}\left({{\varvec{v}}}_{0}\frac{\partial }{\partial {\varvec{y}}}\right)\frac{\partial {\varvec{u}}}{\partial {\varvec{x}}}\right),$$

34 $${\tau }_{yy}= 0 ,$$

whereas, variable thermal conductivity takes the form:

35 $$K\left(\theta \right)=\left(1+\alpha \theta \right).$$

Wall surface geometry expressed as:

36 $$h=1+\varepsilon \mathrm{sin}\left(2\pi x\right).$$

The non-dimensional boundary conditions are:

37 $$\frac{\partial {\varvec{u}}}{\partial {\varvec{y}}}=0\; {\varvec{at}}{\varvec{y}}=0,$$

38 $${\varvec{u}}=-1-\frac{{\varvec{\gamma}}}{(1+{{\varvec{\uplambda}}}_{1})}\frac{\partial {\varvec{u}}}{\partial {\varvec{y}}}\; {\varvec{at}}\; {\varvec{y}}={\varvec{h}},$$

39 $$\frac{\partial{\varvec{\theta}}}{\partial {\varvec{y}}}=0\; {\varvec{at}}\; {\varvec{y}}=0,$$

40 $${\varvec{\theta}}+{{\varvec{\gamma}}}_{1}\frac{\partial{\varvec{\theta}}}{\partial {\varvec{y}}}=0\; {\varvec{at}}\; {\varvec{y}}={\varvec{h}},$$

41 $$\frac{\partial{\varvec{\phi}}}{\partial {\varvec{y}}}=0\; {\varvec{at}}\; {\varvec{y}}=0,$$

42 $${\varvec{\phi}}+{{\varvec{\gamma}}}_{2}\frac{\partial{\varvec{\phi}}}{\partial {\varvec{y}}}=0\; {\varvec{at}}\; {\varvec{y}}={\varvec{h}},$$

where $\gamma , {\gamma }_{1}, {\gamma }_{2}$ are velocity, temperature and concentration slip parameters respectively. The heat transfer at the walls is expressed as:

43 $$Z\left(x\right)={\theta }_{y}\left[h\right]{h}_{x} .$$

In the fixed frame, the instantaneous ow rate is given by^58^:

44 $$\overline{Q }={\int }_{0}^{h}\overline{U }\left(\overline{X },\overline{Y },\overline{t }\right)d\overline{Y }.$$

In a wave frame Eq. (42) takes the form^58^:

45 $$q={\int }_{0}^{h}\overline{u }\left(\overline{x },y\right)d\overline{y }.$$

Over one period of the peristaltic wave, the average non-dimensional volume flow rate $\overline{Q }$ is defined as^58^:

46 $$Q=q+\frac{1}{T}{\int }_{0}^{T}\overline{Q }dt=q+1.$$

## Solution procedure

Since the equations established in the previous section are coupled and nonlinear, they cannot be precisely solved. As a result, exact solutions to these equations cannot be found. But as technology has advanced, a variety of built-in software programs have emerged that can offer the best numerical approximation to such system of equations especially, in confined domains where a maximum to minimum range is attainable. In this method, the entire system is added to obtain a direct graphical illustration of the problem under consideration. Such a method has the advantage of offering a level of accuracy with the least amount of CPU time (5–25 min) every evaluation. One such example is the problem under consideration, which may be solved using the built-in command NDSolve in the computer program MATHEMATICA which provide graphical output with the necessary boundary conditions. Since, the technique is based on shooting method which shows good efficiency for boundary value problem. However, the technique automatically choose starting initial condition which may create problem. As having good starting point become crucial for finding the best solution. Since, this problem can be fixed after choosing appropriate initial point for starting the calculations. Therefore, various researchers have adopted the technique to solve and justify the obtained solutions (see refs.^28,30^).

## Results and discussion

The purpose of the current part is to thoroughly examine how embedded parameters affect flow quantities. For some parameter values, the study that was conducted is useful. Results are discussed for fixed values of $x = 0.3$ and $\varepsilon = 0.1$. Whereas, the other parameters range is taken as, $0\le k\le 0.5$, $0\le H\le 2.0$, $0\le {\lambda }_{1}\le 1.5$, $0\le \alpha \le 0.5$, $0\le Pr\le 2.0$, $0\le B\le 1.0$, $0\le Sc\le 0.5$, $0\le \xi \le 3.0$, $0\le \Omega \le 2.0$, $0\le {\gamma ,\gamma }_{1} ,{\gamma }_{2} \le 0.3$ and $0\le E\le 2.0$.

### Thermal analysis

**Figure 2:**
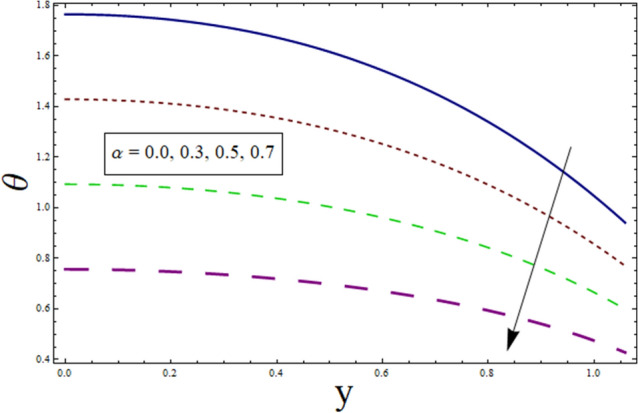
Temperature presentation of α.

**Figure 3:**
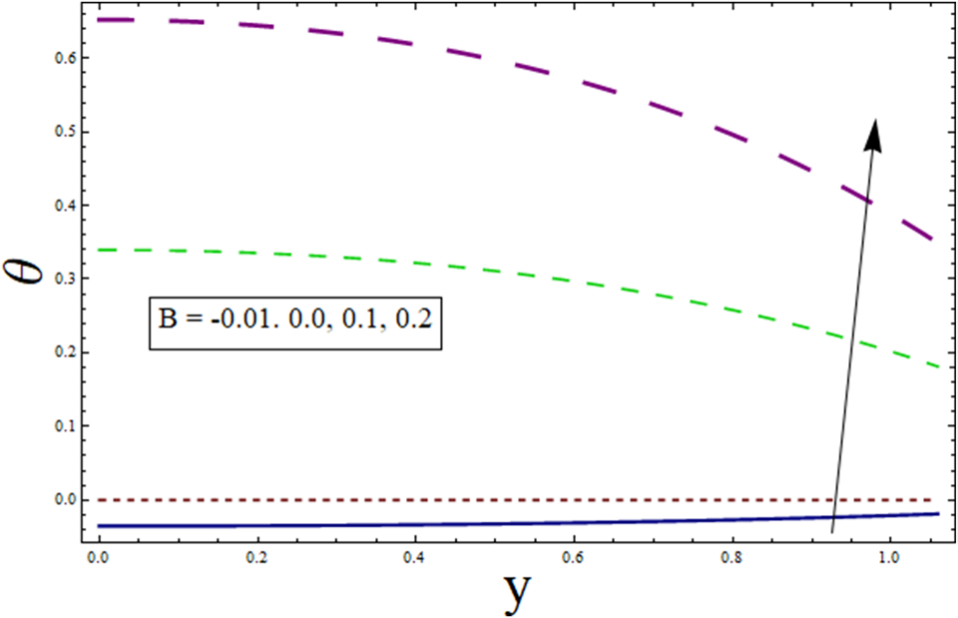
Temperature presentation of B.

**Figure 4:**
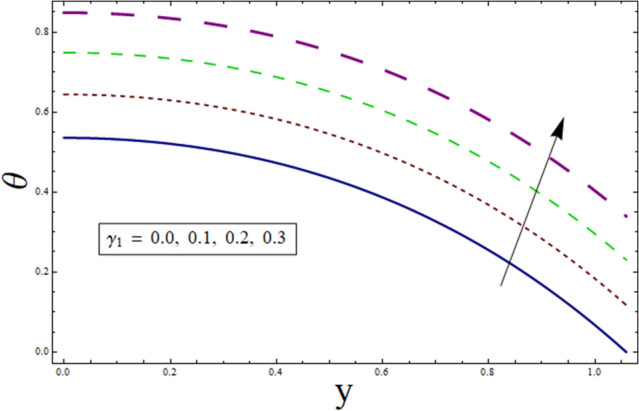
Temperature presentation of ${\gamma }_{1}$.

**Figure 5:**
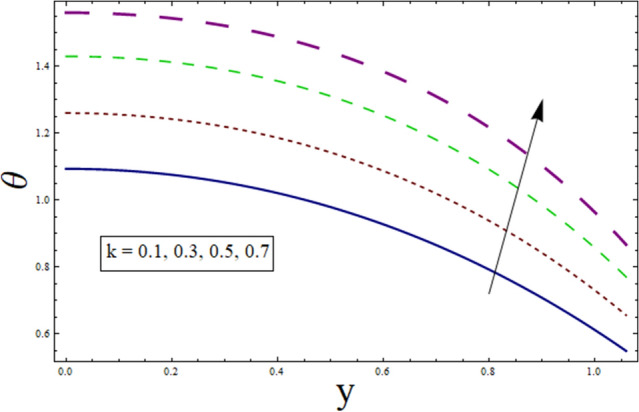
Temperature presentation of $k$.

**Figure 6:**
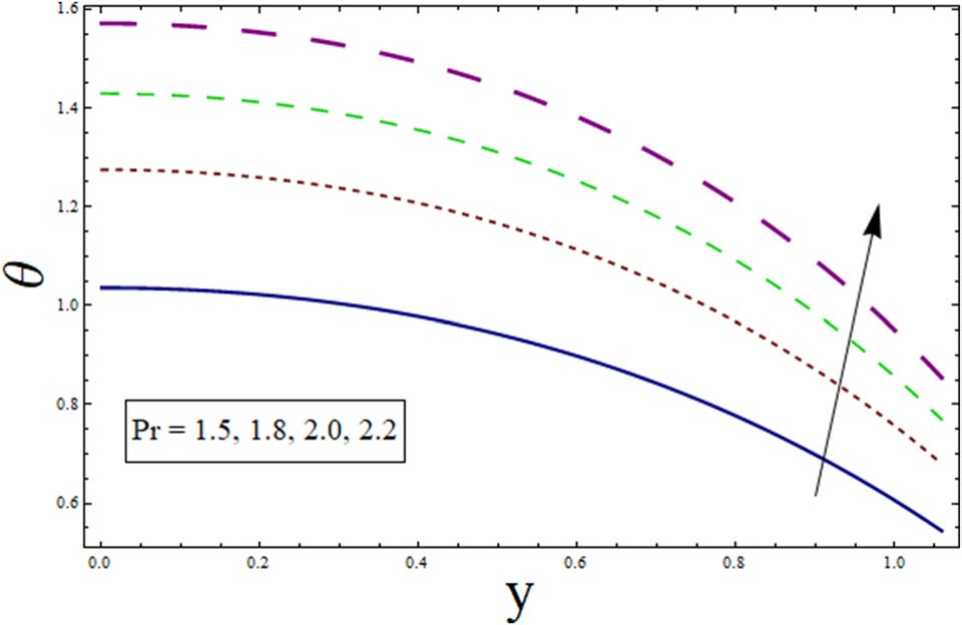
Temperature presentation of $Pr$.

This subsection is prepared to analyze the impact of various parameters on temperature $\theta$. Figure 2 discloses the effect of variable thermal conductivity parameter $\alpha$ on $\theta$. It can be seen that temperature decreases for higher values of $\alpha$. This is due to the fact that material ability to absorb or disperse heat is enhanced for greater values of $\alpha$. To study impact of $B$ on $\theta$, Fig. 3 is plotted. The figure shows the enhanced temperature for increasing the values of $B$. Such enhancement in temperature is caused by the heat generation resulted from the friction between fluid layers. Figure 4 depicts the behavior of thermal slip parameter ${\gamma }_{1}$ on $\theta$. Temperature rises for greater values of ${\gamma }_{1}$. The reason behind this increase can be linked with velocity. As temperature is the average kinetic energy of particles. Increasing slip will result in increased velocity hence, accelerating fluid flow. Therefore, temperature rises. Similar behavior is shown by suction/injection parameter $k$ (see Fig. 5). Increase in the values of $k$ results in large pores. In turn, more fluid will flow through them resulting in higher velocity. Since, temperature is the average kinetic energy of particles. Therefore, temperature enhances. Figure 6 shows the impact of $Pr$ on $\theta$. Rise in temperature is noticed for higher values of $Pr$. Given that Prandtl is the ratio of momentum to thermal diffusivity. Enhancing Prandtl number causes increase in internal friction among fluid layers. Therefore, a higher $Pr$ indicates a higher temperature.

### Velocity analysis

**Figure 7:**
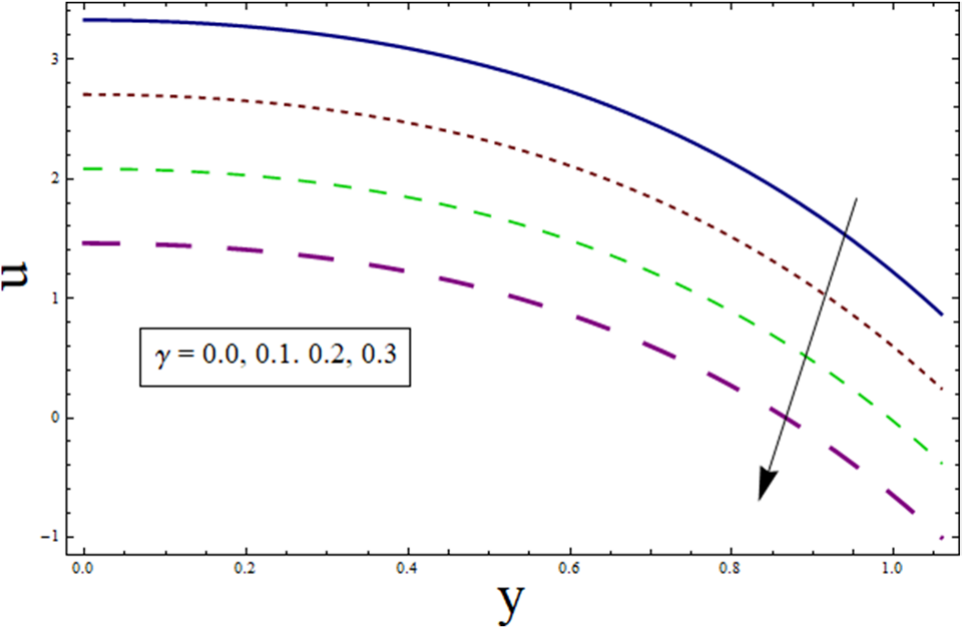
Velocity presentation of $\gamma$.

**Figure 8:**
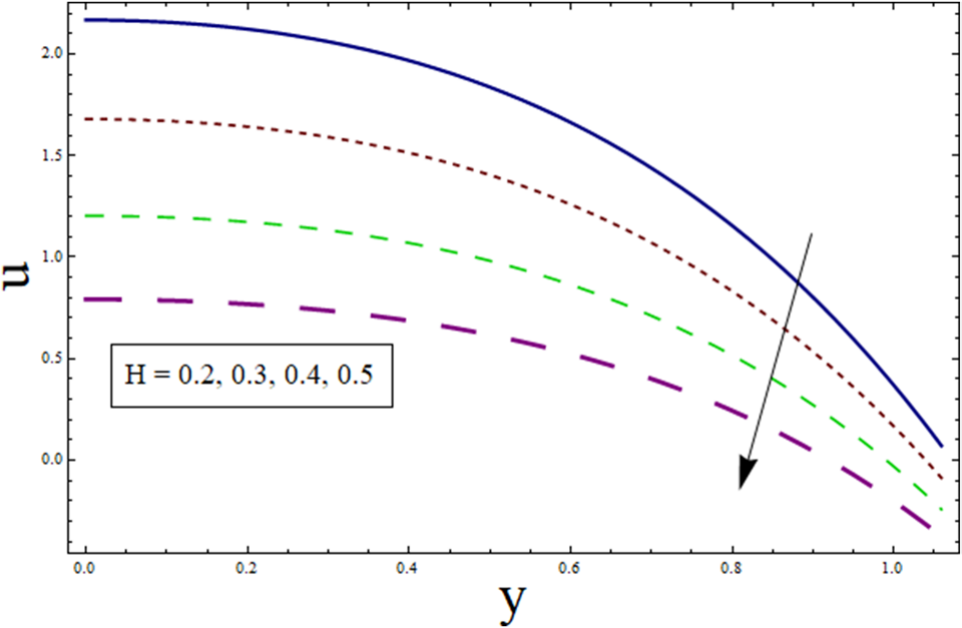
Velocity presentation of $H$.

**Figure 9:**
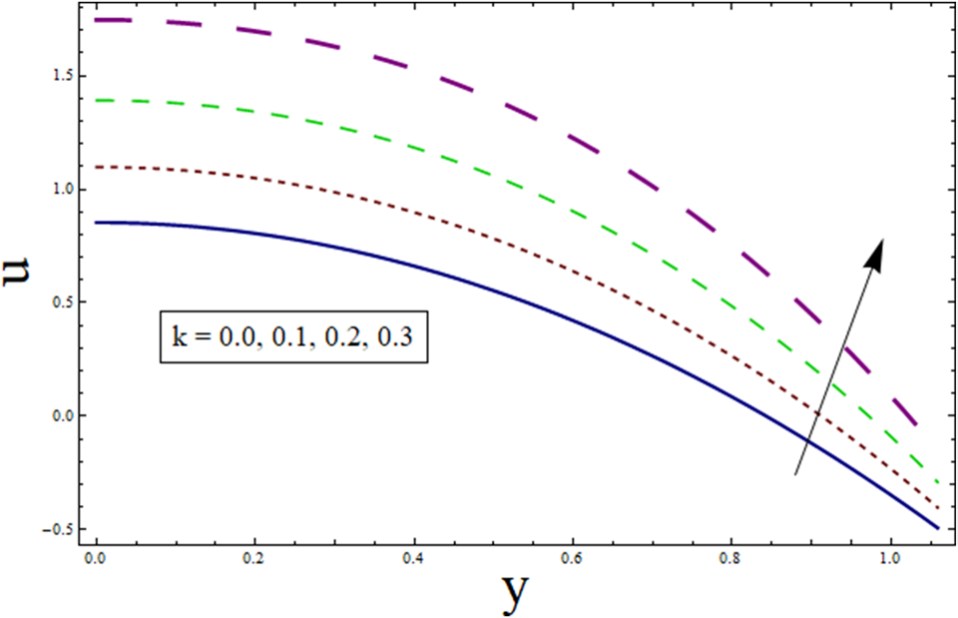
Velocity presentation of $k$.

**Figure 10:**
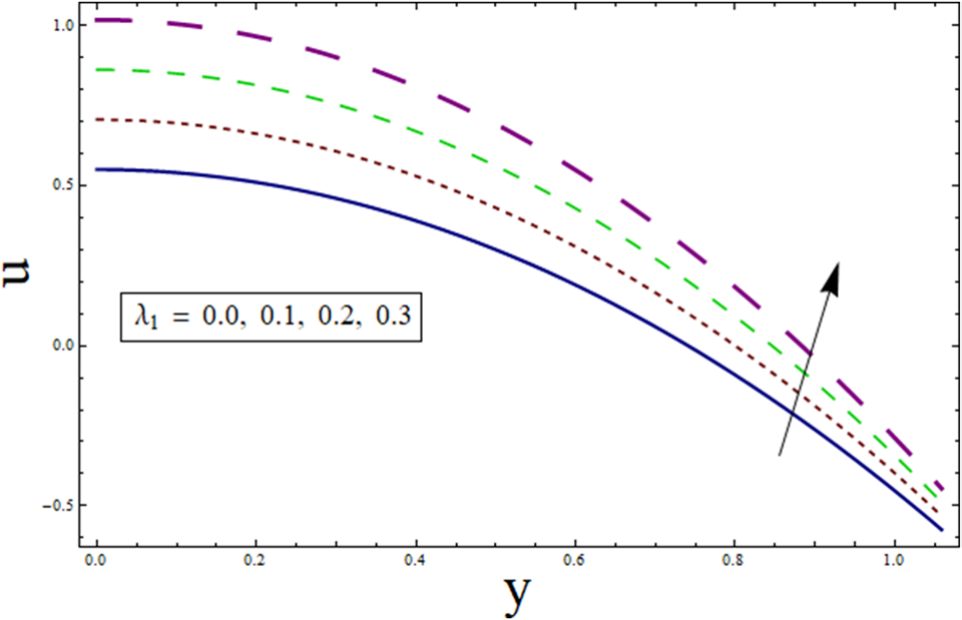
Velocity presentation of ${\lambda }_{1}$.

This subsection analysis the behavior of involved parameters on velocity $u$. Figure 7 shows the effect of slip parameter on $u$. Velocity declines for higher values of $\gamma$. This is due to the fact that as the slippage occurs on the boundary, velocity become different from the boundary. Which, in turn, decreases the effect of boundary on the motion of fluid, resulting in decreased velocity. Figure 8 portrays the significance of Hartman number $H$ on $u$. Since, magnetic field is the resistive kind of force. Therefore, it decreases velocity. Figure 9 discusses the impact of $k$ on $u$. Greater the value of suction/injection parameter, larger the number of pores, thus more fluid flow results. Fluid parameter ${\lambda }_{1}$ effects the velocity in increasing manner. Since it is viscoelastic parameter. Therefore increase in ${\lambda }_{1}$ always results in increased elastane causing fluid velocity to increase. (see Fig. 10).

### Heat transfer analysis

**Figure 11:**
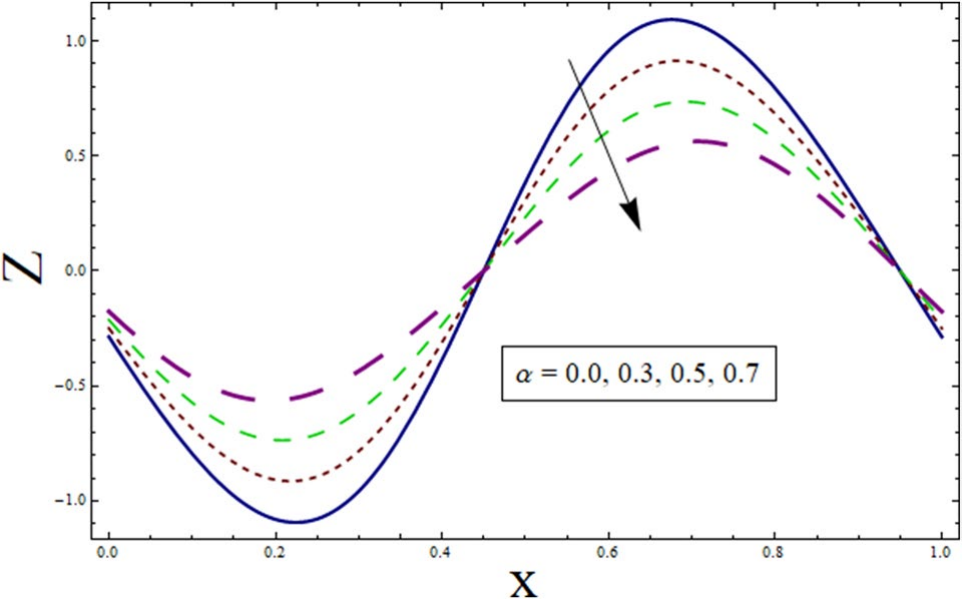
Heat transfer presentation of $\alpha$.

**Figure 12:**
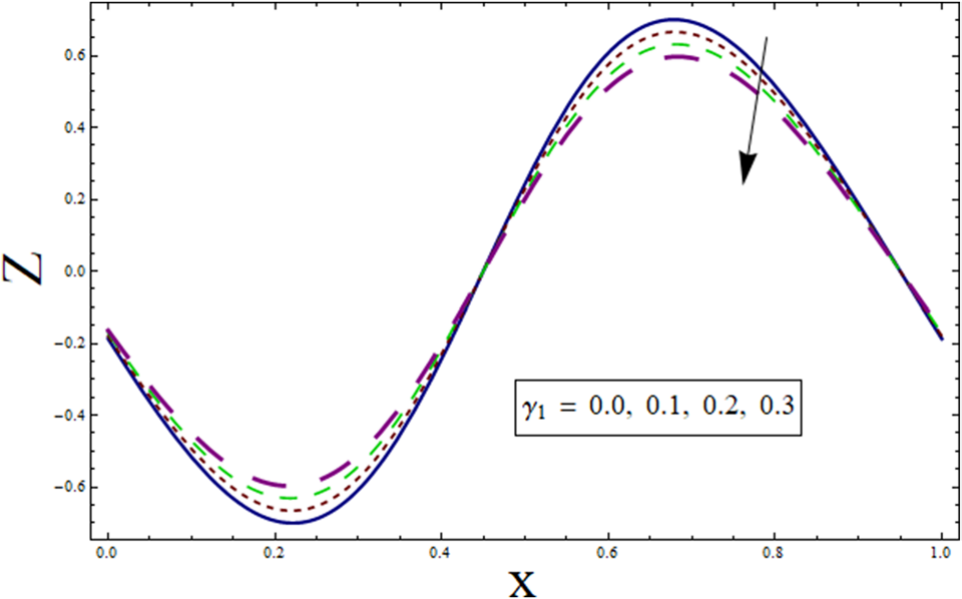
Heat transfer presentation ${\gamma }_{1}$.

**Figure 13:**
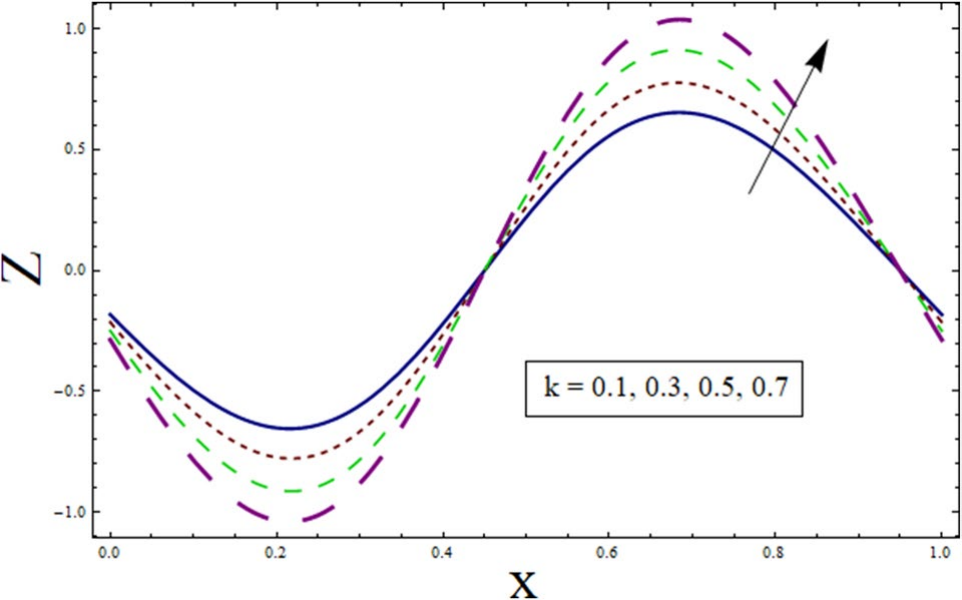
Heat transfer presentation $k$.

**Figure 14:**
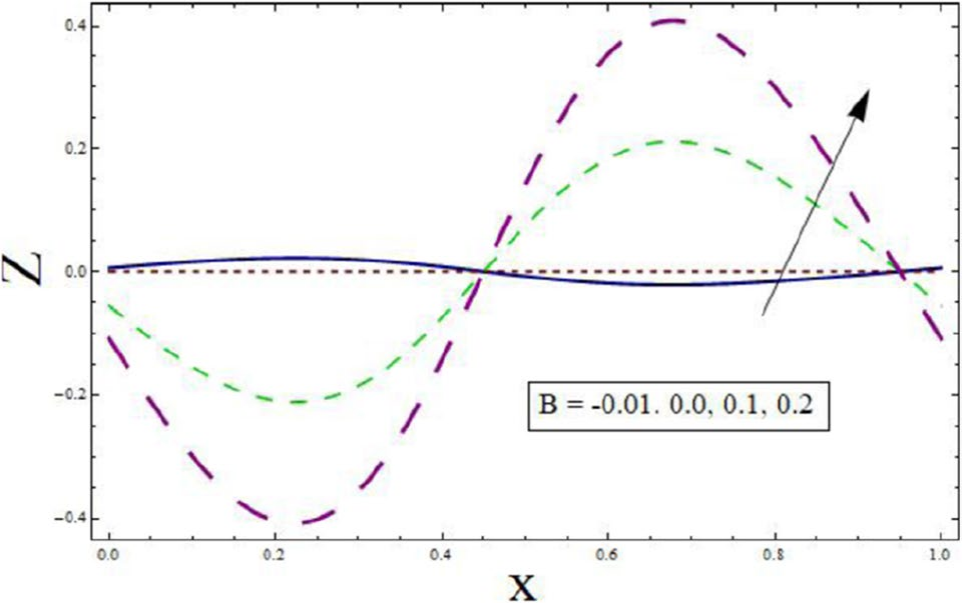
Heat transfer presentation $B$.

**Figure 15:**
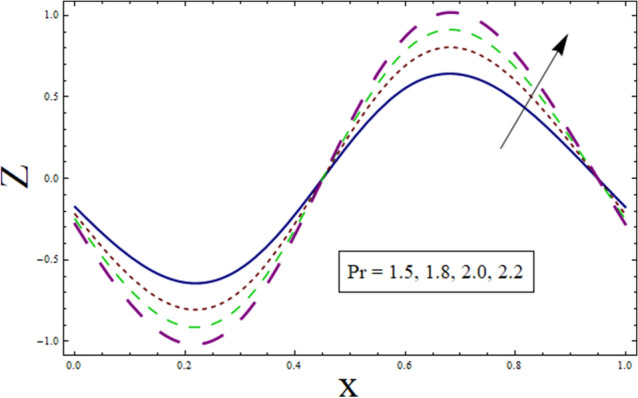
Heat transfer presentation $Pr$.

Through the energy equation, the heat transfer phenomena has a significant impact on the peristalsis process. Therefore, heat transmission considerations are necessary for physiological and industrial applications. Thus, Figs. 11, 12, 13, 14 and 15 study the effect of various parameters on heat transfer profile. It is noticed from the figures that heat transfer shows sinusoidal behavior. This is due to fact that waves at the boundary are of sinusoidal nature due to the consideration of peristaltic waves. Figures 11 and 12 depict the behavior of variable thermal conductivity parameter α and thermal slip parameter ${\gamma }_{1}$. It is noticed from the figures that magnitude of heat transfer decreases for enhancing values of α and ${\gamma }_{1}$. Since increasing the value of α reduces the ability of fluid to absorb heat. Thus, resulting in decrease heat transfer rate. Also, the presence of pores at the boundaries greatly effects the fluid velocity which is directly related with temperature. Therefore, decrease in temperature results in less heat transfer. Figure 13 is sketched to study the effect of suction/injection parameter on $Z$. It is noticed from the figure that rate of heat transfer decreases as we increase the value of $k$. Because higher number of pores results in increased velocity causing temperature to rise. Thus, resulting in higher rate of heat transfer. As we consider $B$ less than $0$, excessive heat is absorbed resulting in decreased rate of heat transfer. On the other hand, taking $B$ greater than $0$ generates more heat causing higher rate of heat transfer (see Fig. 14). Impact of Prandtl number $Pr$ on heat transfer rate can be depicted through Fig. 15. Since, temperature shows increasing trend for $Pr$, thus have higher rate of heat transfer.

### Pressure gradient and pressure rise

**Figure 16:**
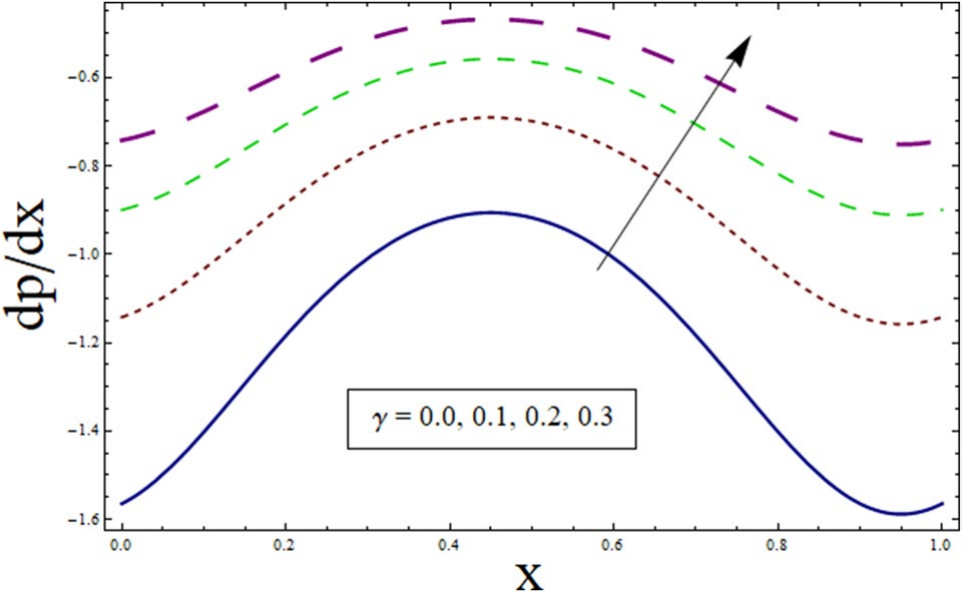
dp/dx for $\gamma$.

**Figure 17:**
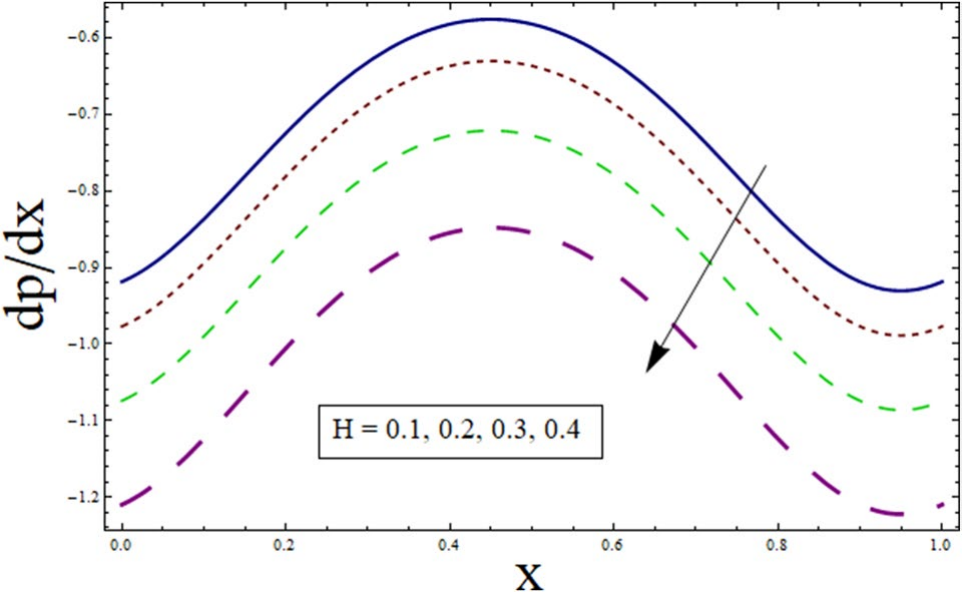
dp/dx for $H$.

**Figure 18:**
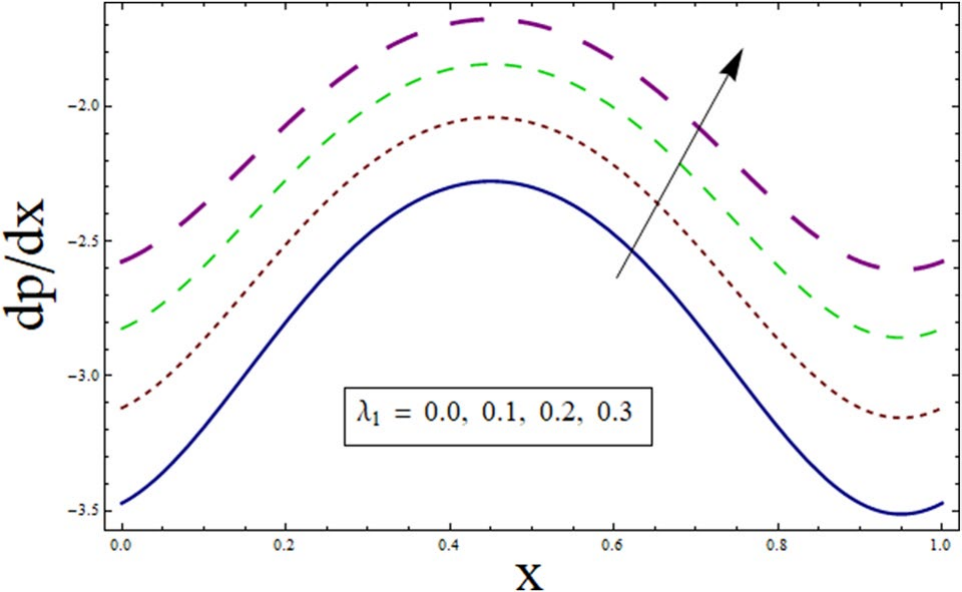
dp/dx for ${\lambda }_{1}$.

**Figure 19:**
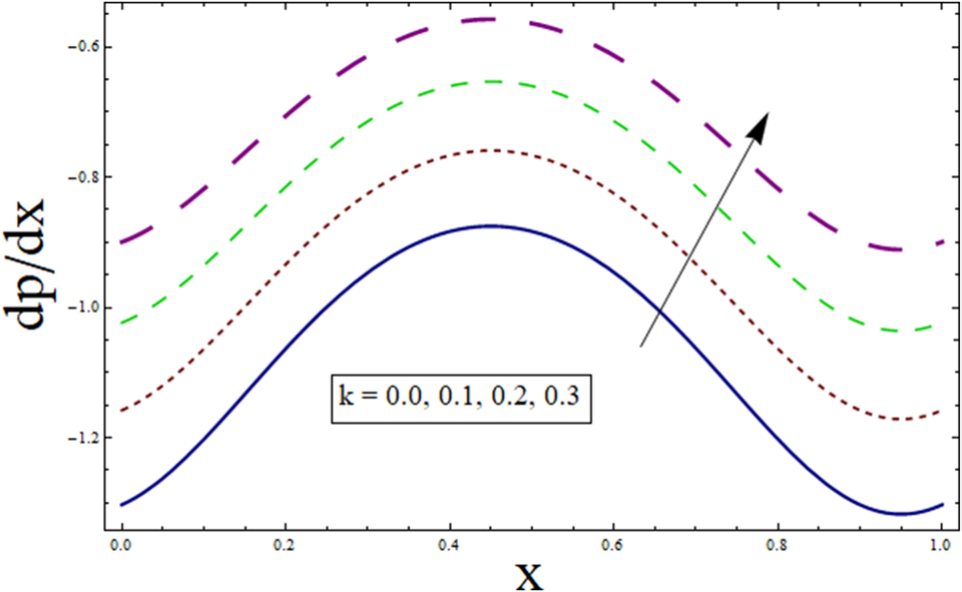
dp/dx for $k$.

**Figure 20:**
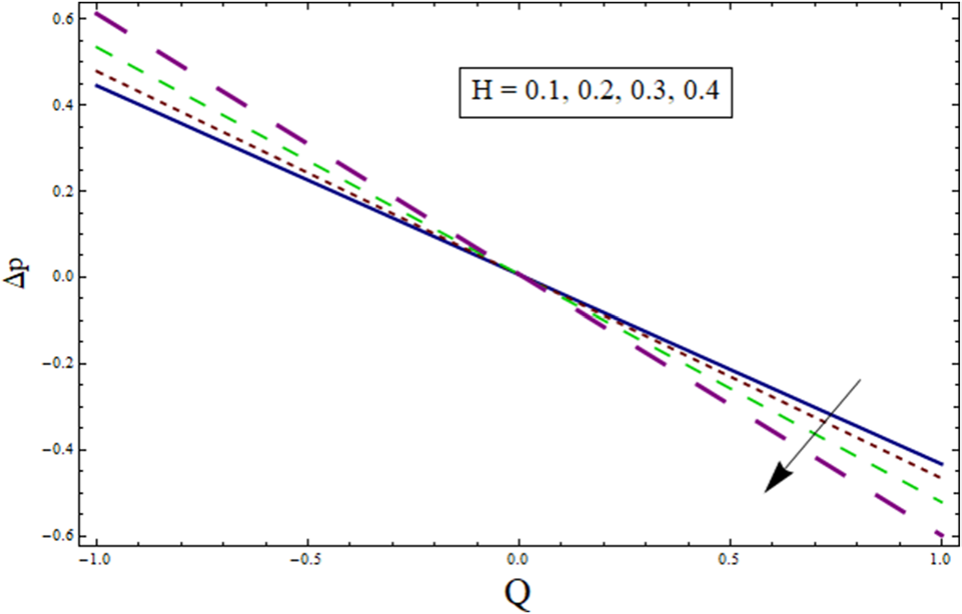
Δp for $$H$$.

**Figure 21:**
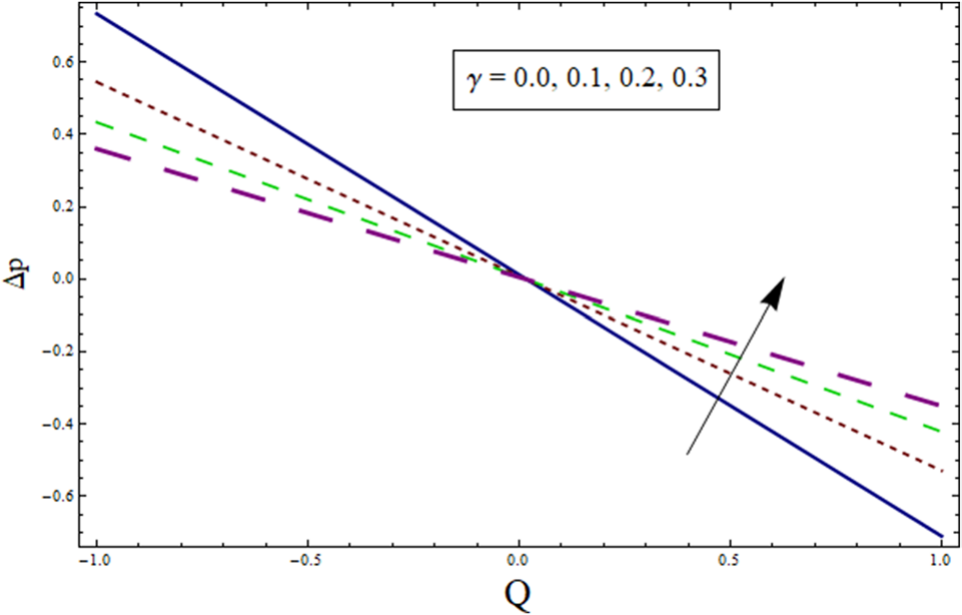
Δp for $$\gamma$$.

**Figure 22:**
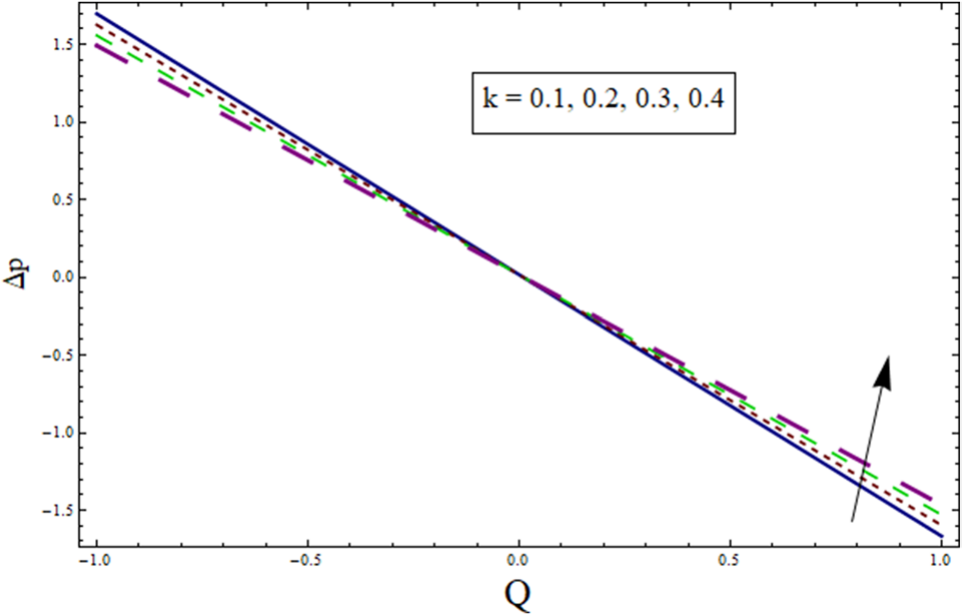
Δp for $$k$$.

**Figure 23:**
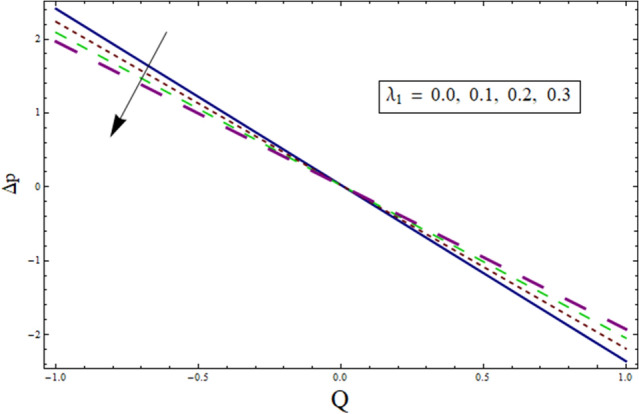
Δp for $${\lambda }_{1}$$.

The axial pressure gradient expressed in terms of independent variable $x$ is plotted for various corresponding parameters. Fluctuating behavior is shown by pressure gradient attaining its minimum at (0.0…, 0.95…), whereas approaching maximum at 0.5 …. This shows the existence of high level flow through the tube without the need of greater pressure gradient. The reason behind fluctuating behavior of pressure gradient is the peristaltic wave traveling along the boundary. For dealing with the results for pressure gradient and pressure rise Figs. 16, 17, 18, 19, 20, 21, 22 and 23 are plotted. For velocity slip, $dp/dx$ enhances, whereas, similar behavior is noticed for Jeffrey fluid parameter ${\lambda }_{1}$ (see Figs. 16 and 18). The higher values of Hartman number $H$ results in greater pressure gradient (see Fig. 17). Figure 19 studies the impact of suction/injection parameter on $dp/dx$. It is observed that pressure gradient rises for $k$.

Additionally, pressure rise for one wavelength is used to examine the maximum pressure rise at which peristalsis acts as a pump. Non-Newtonian fluid characteristics can easily be described by nonlinear nature of these curves. All these figures are composed of four primary sections: the peristaltic pumping zone $(\Delta P > 0)$, the free pumping region $(\Delta P=0)$, and the co-pumping region $(\Delta P< 0)$. Peristalsis, which occurred as a result of pressure difference, causes flow rate to be positive in the zone of peristaltic pumping, whereas peristalsis of the channel boundaries produces a free-pumping region. Negative pressure difference helps the peristalsis-related flow in the co-pumping zone. The effect of $H$ on $\Delta p$ is studied through Fig. 20. It is noticed from the figure that making increment in $H$ results in decrease of $\Delta p$ in co-pumping region. Figure 21 depicts the effect of on pressure rise. Increase in $\Delta p$ can be seen in co-pumping region whereas shows decrease in $\Delta p$ in free pumping region. Figure 22 depicts the impact of suction/injection parameter $k$ on $\Delta p$. It is noticed that pumping curves meet at point $Q\approx 0.1$. For $Q>0.1$, pressure rise enhances whereas opposite behavior is noticed for $Q<0.1$. Figure 23 shows the effect ${\lambda }_{1}$ of on $\Delta p$. Pumping rate is found to be enhancing in co-pumping region whereas shows opposite behavior in free pumping region.

### Concentration profile

**Figure 24:**
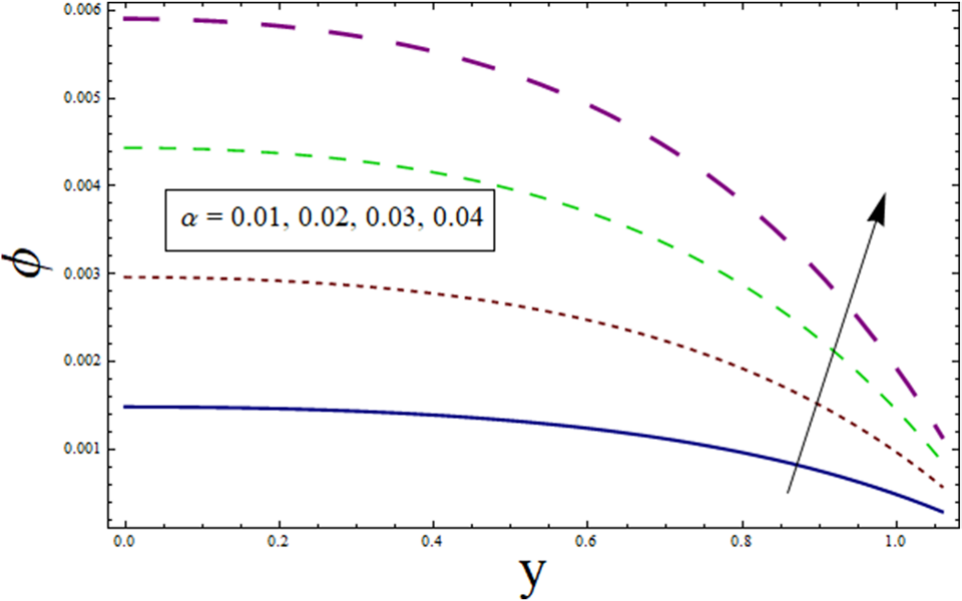
ϕ for $$\alpha$$.

**Figure 25:**
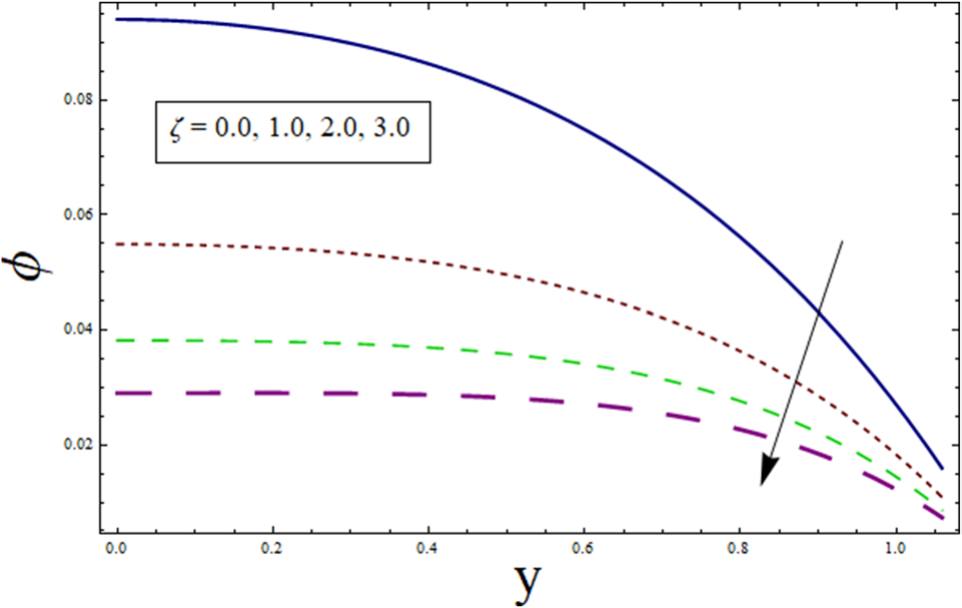
ϕ for $$\xi$$.

**Figure 26:**
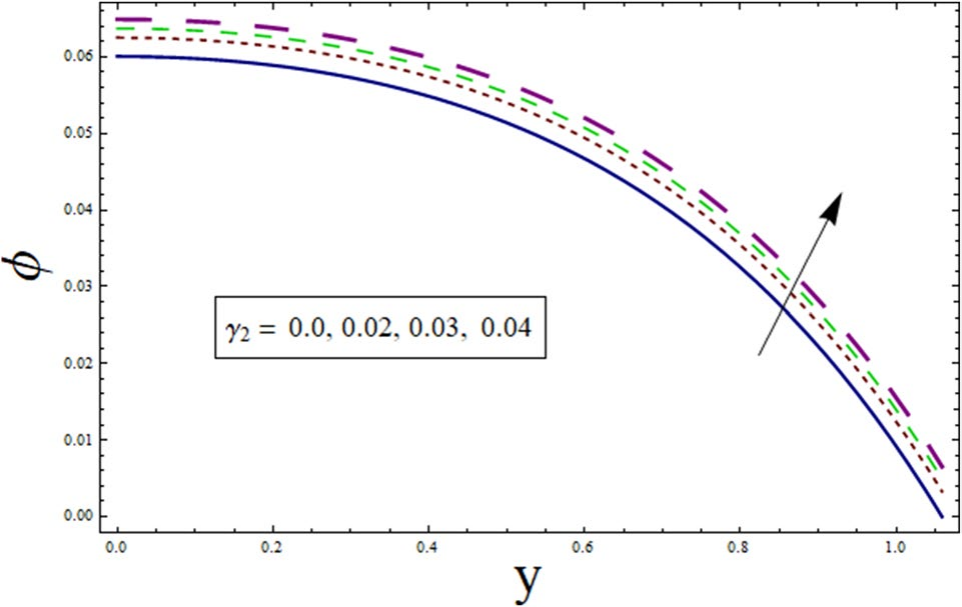
ϕ for $${\gamma }_{1}$$.

**Figure 27:**
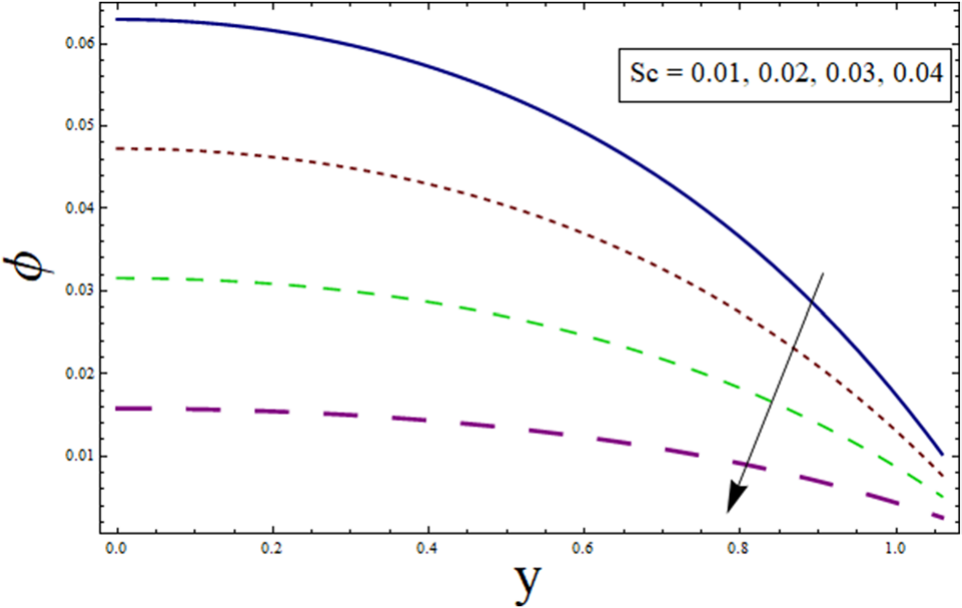
ϕ for $$Sc$$.

**Figure 28:**
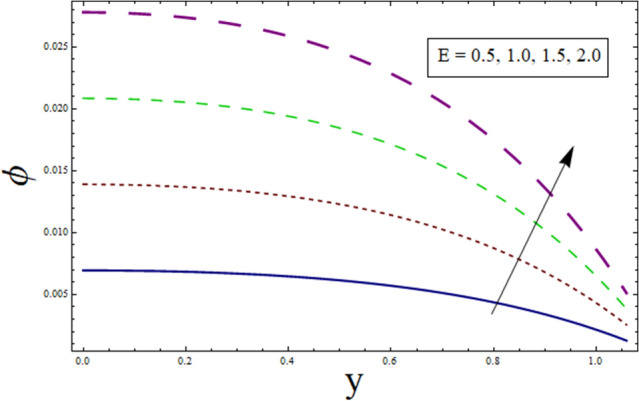
ϕ for $$E$$.

**Figure 29:**
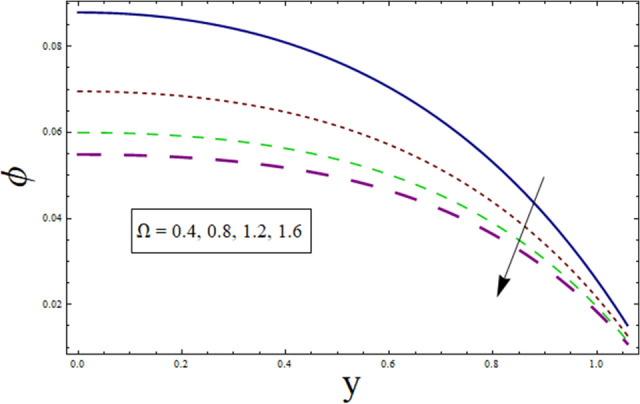
ϕ for $$\Omega$$.

This subsection addresses the effect of various parameters on concentration profile $\phi$. The concentration profiles exhibit the opposite behavior from the temperature profiles. Physically speaking, this makes sense because heat and mass are known to be opposites. Additionally, the patterns show that the fluid particles are more concentrated close to the channel walls. For this purpose Figs. 24, 25, 26, 27, 28 and 29 are plotted. It can be analyzed through Fig. 24 that concentration increases for higher values of variable thermal conductivity parameter $\alpha$. Since variation in thermal conductivity of material greatly influence the temperature, thus effecting concentration. Figure 25 depicts the impact of $\xi$ (rate of chemical reaction) on ϕ. The concentration is decreasing function of $\xi$. To elucidate the effect of concentration slip parameter ${\gamma }_{2}$, Fig. 26 is plotted. Concentration increases for higher values of ${\gamma }_{2}$. To demonstrate the effect of Schmidt number $Sc$ on $\phi$, Fig. 27 is sketched. Enhancement in the values of $Sc$ decreases $\phi$. Increase in $Sc$ causes concentration diffusion to decrease, hence resulting in less concentration at the point. Impact of activation energy $E$ can be depicted through Fig. 28. It shows that concentration enhances for higher values of $E$. Figure 29 elucidates the effect of temperature ratio parameter . Decrease is noticed for concentration when values of Ω are enhanced.

## Conclusions

The current study is performed to analyze the 2-dimensional peristaltic flow through porous walled channel in the presence of magnetic field. Energy equation is modelled by considering heat source/sink parameter. Whereas, activation energy is considered for the analysis of concentration profile. In addition, slip conditions are imposed to study flow quantities. The main outcomes of the analysis are:

1. Velocity shows parabolic behavior near the center of channel.
2. Decreasing behavior is noticed for slip parameter $\gamma$ whereas enhancement is noticed for $k$ and ${\lambda }_{1}$.
3. Similar profile is exhibited by temperature and velocity.
4. Temperature is enhanced for $B$ and ${\gamma }_{1}$ whereas decreases for $\alpha$.
5. Heat transfer and pressure gradient are of sinusoidal nature.
6. Pressure rise shows increasing behavior for $\gamma , k$ and ${\lambda }_{1}$ in co-pumping region.
7. Concentration depicts increase for higher values of ${\gamma }_{2}$ and α whereas show decrease for $Sc$ and $\xi$.

The study presented in this article finds interesting application in various human physiological systems. Majority organs within human body show porous nature. In addition, presence of fluids make boundaries slippery in nature. Thus, we can say that the mathematical model developed in current analysis can be used to predict functioning of variety of systems. From future perspective, addition of nanoparticles, consideration of viscous dissipation with thermal radiations can be taken as mathematical model to study the treatment of cancer within any physiological system. Also, taking convective conditions at the boundaries in addition to Soret/Dufour effects may help to develop model for thermal analysis of digestive system. Thus, in the culmination of above analysis peristalsis finds numerous applications in multiple disciplines.

## Data Availability

The data used to support the findings of this study are available from the corresponding author upon request.

## List of symbols

$\overline{H }/h$: Dimensional/non-dimensional wall surface
$$\overline{t }$$: Time
$$\lambda$$: Wavelength
$a, b$: Width of the channel, wave amplitude
$c$: Speed of wave
$$\left(\overline{X }, \overline{Y }\right)$$/$$\left(x,y\right)$$: Cartesian coordinates
$\left(\overline{U }, \overline{V }\right)/\left(u, v\right)$: Dimensional/non-dimensional velocity components along horizontal and vertical directions
$\overline{{V }_{0}},{v}_{0}$: Dimensional/non-dimensional suction/injection velocity
$$\rho$$: Fluid density
$$\overline{P }$$: Pressure
$\sigma /{B}_{0}$: Electrical conductivity/magnetic field strength
$$\tau$$: Stress tensor
${C}_{p}$: Specific heat capacity
$\overline{T }/\theta$: Dimensional/non-dimensional Temperature
$\overline{K }\left(\overline{T }\right)$: Variable thermal conductivity
${Q}_{0}$: Constant heat source/sink parameter
$\overline{C }/\phi$: Dimensional/non-dimensional concentration
$D$: Mass diffusion coefficient
${K}_{1}$: Chemical reaction parameter
$${K}_{2}$$: Reaction rate
${E}_{a}/E$: Dimensional/non-dimensional activation energy
$n$: Fitted rate constant
$${K}^{*}$$: Boltzman constant
${K}_{m}$: Thermal conductivity at constant temperature
α: Constant
$\overline{\gamma }$, $\overline{{\gamma }_{1}}$,$\overline{{\gamma }_{2}}$/$\gamma ,{\gamma }_{1},{\gamma }_{2}$: Dimensional/non-dimensional velocity, temperature and concentration slip
${\uplambda }_{1}/{\uplambda }_{2}$: Jeffrey fluid parameters
$$\delta$$: Wave number
$$Re$$: Reynolds number
$$H$$: Hartman number
$$Pr$$: Prandtl number
$B$: Heat source/sink parameter
$$Sc$$: Schmidt number
${K}_{c}$: Chemical reaction parameter
$\Omega$: Temperature ratio parameter
$\varepsilon$: Dimensionless wave amplitude
$Z$: Heat transfer rate
$\overline{Q }$: Instantaneous flow rate in fixed frame
$q$: Flow rate in wave frame
